# Supplementary material for: Abscisic Acid Metabolizing Rhodococcus sp. Counteracts Phytopathogenic Effects of Abscisic Acid Producing Botrytis sp. on Sunflower Seedlings
Source: Plants (Basel). 2025 Aug 7;14(15):2442. doi: 10.3390/plants14152442 (PMC12349427; doi:10.3390/plants14152442)
Supplement: Supplementary file 1 [file plants-14-02442-s001.zip › plants-3772258-supplementary.pdf]

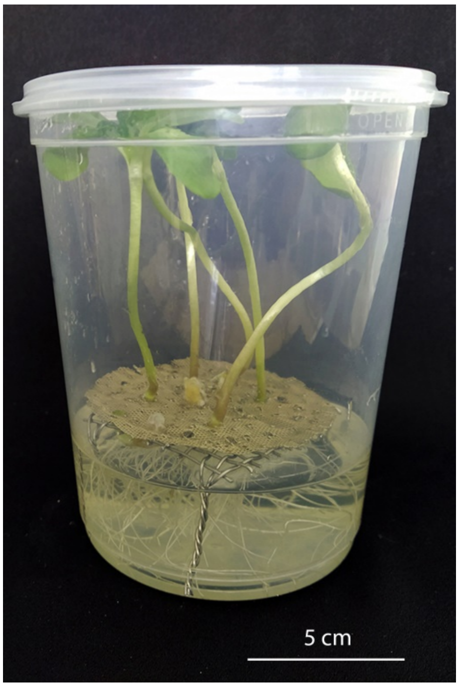

Figure S1. An example of pot with sunflower plants at the day of harvesting.

Table S1. The percentage of query coverage, similarity, and accession numbers for the top ten fungal type strains related to the BA3 strain, based on BLASTn searches in the NCBI GenBank using internal transcribed spacer (ITS) gene sequences.

Type strain	Query Coverage (%)	Similarity (%)	Accession
Botrytis eucalypti CERC 7170	98	100	KX301016.1
Botrytis pelargonii CBS 497.50	90	100	AJ716290.1
Botrytis fabae CBS 120.29	90	100	MH855020.1
Botrytis caroliniana ATCC MYA-4856	97	99.80	NR_111839.1
Botryotinia ranunculi CBS 178.63	100	99.61	NR_164278.1
Botrytis californica WSP 72753	100	99.61	NR_151843.1
Botrytis deweyae CBS 134650	100	99.61	NR_171711.2
Botrytis sinoallii OnionBC-23	100	99.61	EU519203.1
Botryotinia polyblastis CBS 287.38	90	99.57	NR_164208.1
Botrytis porri CBS 190.26	100	99.02	MH854885.1

Table S2. Correlation coefficients between concentrations of phytohormones in sunflower and the effect on plant biomass of all the treatments applied relative to uninoculated and untreated control (n = 6).

Parameters	Effect on shoot biomass (%)	Effect on root biomass (%)	Shoot / Root ratio
Root ABA	-0.9563<br>$p < 0.001$	-0.9388<br>$P = 0.001$	-0.0771<br>$p = 0.856$
Root SA	0.0337<br>$p = 0.937$	-0.1019<br>$p = 0.810$	0.3640<br>$p = 0.375$
Root Ja	-0.6738<br>$p = 0.067$	-0.5605<br>$p = 0.148$	-0.3214<br>$p = 0.438$
Root IAA	0.2287<br>$p = 0.586$	0.4583<br>$p = 0.253$	-0.6296<br>$p = 0.094$
Root GA3	-0.3365<br>$p = 0.415$	-0.0852<br>$p = 0.841$	-0.6695<br>$p = 0.069$
Root DHZ	-0.4595<br>$p = 0.252$	-0.2656<br>$p = 0.525$	-0.5046<br>$p = 0.202$
Root tZ	0.3357<br>$p = 0.416$	0.3220<br>$p = 0.437$	0.0264<br>$p = 0.951$
Root tZR	-0.7980<br>$p = 0.018$	-0.6879<br>$p = 0.059$	-0.2875<br>$p = 0.490$
Shoot ABA	0.2032<br>$p = 0.629$	0.0927<br>$p = 0.827$	0.3153<br>$p = 0.447$
Shoot SA	0.1726<br>$p = 0.683$	0.0018<br>$p = 0.997$	0.4617<br>$p = 0.249$
Shoot Ja	0.2982<br>$p = 0.473$	0.2304<br>$p = 0.583$	0.2463<br>$p = 0.556$
Shoot IAA	0.1158<br>$p = 0.785$	-0.0268<br>$p = 0.950$	0.4444<br>$p = 0.270$
Shoot GA3	-0.3006<br>$p = 0.469$	-0.4614<br>$p = 0.250$	0.4912<br>$p = 0.216$
Shoot DHZ	-0.1230<br>$p = 0.772$	-0.1981<br>$p = 0.638$	0.1927<br>$p = 0.648$
Shoot tZ	0.4852<br>$p = 0.223$	0.3044<br>$p = 0.463$	0.5073<br>$p = 0.199$
Shoot tZR	0.0397<br>$p = 0.926$	-0.1152<br>$p = 0.786$	0.4505<br>$p = 0.263$

Red values indicate significant correlation.

Table S3. Correlation coefficients between concentrations of phytohormones in sunflower plants (n = 6).

Parameters	Root ABA	Root SA	Root JA	Root IAA	Root GA3	Root DHZ	Root tZ	Root tZR	Shoot ABA	Shoot SA	Shoot JA	Shoot IAA	Shoot GA3	Shoot DHZ	Shoot tZ
Root SA	-0.0365														
	p = 0.932														
Root JA	0.6031	0.3949													
	p = 0.113	*p* = 0.333													
Root IAA	-0.1671	-0.3276	-0.1518												
	p = 0.692	*p* = 0.428	*p* = 0.720												
Root GA3	0.2586	-0.6992	0.1801	0.4574											
	p = 0.536	*p* = 0.054	*p* = 0.669	*p* = 0.254											
Root DHZ	0.4533	-0.5840	0.2953	0.4835	0.8467										
	p = 0.259	*p* = 0.128	*p* = 0.478	*p* = 0.225	*p* = 0.008										
Root tZ	-0.3650	-0.0804	-0.3711	0.0362	-0.4101	-0.1986									
	p = 0.374	*p* = 0.850	*p* = 0.365	*p* = 0.932	*p* = 0.313	*p* = 0.637									
Root tZR	0.7083	0.0900	0.8109	-0.0460	0.4709	0.6606	-0.3585								
	p = 00.049	*p* = 0.832	*p* = 0.015	*p* = 0.914	*p* = 0.239	*p* = 0.075	*p* = 0.383								
Shoot ABA	-0.2846	0.5938	0.3306	-0.6515	-0.4402	-0.5826	-0.1526	-0.0645							
	p = 0.495	*p* = 0.121	*p* = 0.424	*p* = 0.080	*p* = 0.275	*p* = 0.130	*p* = 0.718	*p* = 0.879							
Shoot SA	-0.1470	0.7474	0.2585	-0.5586	-0.6017	-0.7260	-0.3471	-0.1898	0.8616						
	p = 0.728	*p* = 0.033	*p* = 0.536	*p* = 0.150	*p* = 0.115	*p* = 0.041	*p* = 0.400	*p* = 0.652	*p* = 0.006						
Shoot JA	-0.3626	0.1003	0.1196	-0.3693	0.1122	-0.0654	-0.4014	0.0505	0.7034	0.4748					
	p = 0.377	*p* = 0.813	*p* = 0.778	*p* = 0.368	*p* = 0.791	*p* = 0.878	*p* = 0.324	*p* = 0.905	*p* = 0.052	*p* = 0.234					
Shoot IAA	-0.1514	0.2830	0.2132	-0.5466	-0.0274	-0.1547	-0.5356	0.1449	0.7394	0.6217	0.9407				
	p = 0.721	*p* = 0.497	*p* = 0.612	*p* = 0.161	*p* = 0.949	*p* = 0.715	*p* = 0.171	*p* = 0.732	*p* = 0.036	*p* = 0.100	*p* = 0.000				
Shoot GA3	0.2627	0.0702	0.0102	-0.3732	0.1219	0.2264	-0.3322	0.4480	-0.0378	-0.0630	0.3005	0.4703			
	p = 0.530	*p* = 0.869	*p* = 0.981	*p* = 0.363	*p* = 0.774	*p* = 0.590	*p* = 0.421	*p* = 0.266	*p* = 0.929	*p* = 0.882	*p* = 0.470	*p* = 0.240			
Shoot DHZ	-0.0791	0.2071	-0.0821	-0.6071	-0.3413	-0.4132	0.4203	-0.0010	0.2515	0.0508	-0.0736	-0.0146	0.2762		
	p = 0.852	*p* = 0.623	*p* = 0.847	*p* = 0.110	*p* = 0.408	*p* = 0.309	*p* = 0.300	*p* = 0.998	*p* = 0.548	*p* = 0.905	*p* = 0.862	*p* = 0.973	*p* = 0.508		
Shoot tZ	-0.5142	0.3614	-0.1921	-0.6184	-0.4853	-0.7650	-0.1525	-0.5044	0.8329	0.8054	0.6624	0.6886	-0.0128	0.2411	
	p = 0.192	*p* = 0.379	*p* = 0.649	*p* = 0.102	*p* = 0.223	*p* = 0.027	*p* = 0.718	*p* = 0.202	*p* = 0.010	*p* = 0.016	*p* = 0.074	*p* = 0.059	*p* = 0.976	*p* = 0.565	
Shoot tZR	-0.1345	0.3405	0.2275	-0.8274	-0.2492	-0.4205	-0.2762	0.0359	0.8917	0.7106	0.7997	0.8702	0.2938	0.3533	0.8211
	p = 0.751	*p* = 0.409	*p* = 0.588	*p* = 0.011	*p* = 0.552	*p* = 0.300	*p* = 0.508	*p* = 0.933	*p* = 0.003	*p* = 0.048	*p* = 0.017	*p* = 0.005	*p* = 0.480	*p* = 0.391	*p* = 0.012

Red values indicate significant correlation.
